# Metabolic Engineering for Gibberellic Acid Production in *Fusarium fujikuroi*: Advances and Perspectives

**DOI:** 10.3390/molecules31132367

**Published:** 2026-07-05

**Authors:** Lianghong Yin, Xiaoxiao Liu, Jiaoya Chen, Nana Ding, Hui Chen, Haiping Lin, Zheng Ma, Qingsong Shao, Dan Wang, Peng Zhang

**Affiliations:** 1 National Key Laboratory for Development and Utilization of Forest Food Resources, Zhejiang A&F University, Hangzhou 311300, China19906838639@163.com (J.C.); dingnana911128@163.com (N.D.); huichen@zafu.edu.cn (H.C.); sqszjfc@126.com (Q.S.); 2 College of Forestry and Biotechnology, Zhejiang A&F University, Hangzhou 311300, China; 133965578050@163.com; 3 Zhejiang Provincial Key Laboratory of Biometrology and Inspection & Quarantine, College of Life Sciences, China Jiliang University, Hangzhou 310018, China

**Keywords:** *Fusarium fujikuroi*, Gibberellic acids, synthetic biology, metabolic engineering

## Abstract

Gibberellic acids (GAs) are a class of tetracyclic diterpene carboxylic acid compounds produced by green plants, fungi, and bacteria, which have a wide range of applications in agricultural production and food ingredients processing. Owing to the continuously growing market demand, enhancing GA yield has become imperative. The biosynthesis of GAs is a multi-enzymatic synergistic process that can be enhanced through genetic and metabolic engineering strategies. In this review, we first summarize recent advances in GA production by *Fusarium fujikuroi*. We then highlight key metabolic engineering strategies, including biosynthetic pathway engineering, cluster-specific channeling of geranylgeranyl diphosphate biosynthesis, cofactor engineering, as well as regulatory mechanisms involving nitrogen modulation and histone modification. Finally, we discuss promising approaches for constructing high-efficiency microbial cell factories, such as implementation of the CRISPR/Cas9 system, the application of strong promoters, the development of target-specific technologies for small molecules, and the employment of genome-scale metabolic models. Recent metabolic engineering efforts have achieved GA3 titers of up to 3.16 g/L through multi-target nitrogen regulation strategies, highlighting the potential for further yield improvement.

## 1. Introduction

Gibberellic acids (GAs) are diterpenoids derived from four isoprene units and synthesized primarily in stems, roots, flowers, and fruits [1], and they have been pivotal in agricultural advances such as the Green Revolution by regulating key developmental processes including seed germination, stem elongation, flowering, fruit set, and other growth-related activities [2,3]. Interestingly, the same fungus is not only the industrial producer of GAs but also the causal agent of bakanae disease in rice, a major seed-borne disease causing substantial yield losses [4,5].

Among the more than 136 known GAs, biologically active forms such as GA3 [6], GA4, and GA7 are commercially utilized in agricultural products, either as pure GAs or in formulated mixtures [7,8]. In contrast to these C19 GAs, a novel C20-type GA with an atypical backbone, designated DHGA12, has also been shown to promote seed germination and seedling growth in *Arabidopsis thaliana* [9]. GAs are highly effective, broad-spectrum plant growth regulators widely applied in agriculture, for example, in hybrid rice seed production, fruit retention in citrus and cotton, and vegetable cultivation [10], and they also enhance yield and quality in food production via physiological regulation, as exemplified in barley malting, where exogenous GA3 promotes hydrolytic enzyme secretion from the aleurone layer, accelerating endosperm reserve mobilization, shortening the malting period, and increasing extract yield [11]. In postharvest citrus, GA3 dipping or spraying delays peel aging, reduces decay, and extends shelf life [12,13]. Beyond GA3, other gibberellin analogues also play distinct roles. In barley, GA1, GA4, and GA7 induce α-amylase, with GA4 being the predominant endogenous form that rises before enzyme activation. In maize, GA4/GA7 improves grain filling by modulating hormones and antioxidants. In pear, GAs regulate cell expansion and lignification via hormone signaling and cell wall genes. These findings highlight the need for crop- and stage-specific GA selection [14,15].

GAs are industrially produced by *F. fujikuroi*, a phytopathogenic mold responsible for rice bakanae disease. Despite a well-characterized biosynthetic pathway, efficient biomanufacturing is hindered by a complex regulatory network and limited genetic tools [16]. The productivity bottlenecks—whether from precursor supply, flux competition, nitrogen regulation, or cofactor availability—remain unclear. For example, the mevalonate pathway supplies isopentenyl diphosphate (IPP), and hydroxymethylglutaryl coenzyme A (HMG-CoA) reductase often limits flux. Sterol/carotenoid pathways may divert farnesyl diphosphate (FPP) from GA biosynthesis. Nitrogen regulators AreA and AreB tightly control GA biosynthetic gene expression, and NADPH availability for P450 monooxygenases may also restrict conversion. Elucidating these constraints is key to strain improvement [17]. Addressing these multifaceted bottlenecks requires an integrated strategy spanning transcriptional regulation, systems-level analysis, and genome editing [18,19].

In this review, we provide a comprehensive overview of established GA biosynthetic pathways and systematically discuss these metabolic engineering approaches aimed at enhancing production yield.

## 2. Gibberellin Biosynthesis in *F. fujikuroi*

**Figure 1:**
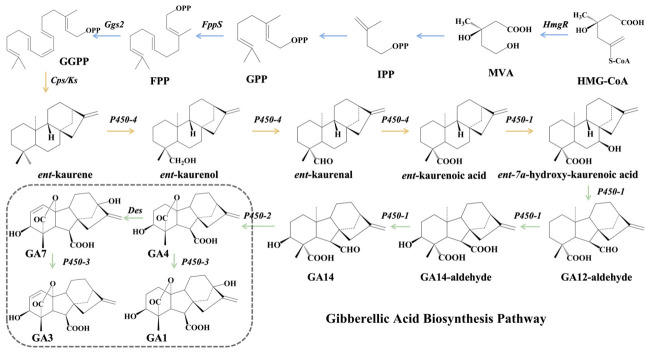
GA biosynthesis pathway.

The biosynthesis of GGPP is the first step of GA synthesis in *F. fujikuroi*, where GGPP is a co-synthetic precursor of diterpenoids [20]. Endogenous acetyl-coenzyme A (acetyl-CoA) [21] is used as a raw material to produce mevalonate (MVA), a precursor of GAs, catalyzed by hydroxymethylglutaryl-coenzyme A reductase (*HmgR*), IPP, and geranyl diphosphate (GPP) [22,23]. *HmgR* is a key regulatory enzyme in the GA biosynthetic pathway [24]. It catalyzes the formation of MVA from HMG-CoA [25,26]. Lovastatin is produced by fermentation of *Aspergillus terreus* as a secondary metabolite. It competitively inhibits *HmgR* [27], and this inhibition can be reversed by exogenous mevalonate, revealing a key feedback node shared between GA and statin biosynthesis. IPP joins with GPP in a head-to-tail fashion to produce FPP, and IPP joins with FPP to form GGPP, a common precursor of the diterpenes [21,28] (Figure 1).

GGPP, which is synthesized by geranylgeranyl diphosphate synthase 2 (*Ggs2*), is subsequently converted to ent-kaurene via ent-copalyldiphosphate in a two-step cyclization reaction catalyzed by the diterpene cyclase copalyldiphosphate synthase/kaurene synthase (*Cps/Ks*). The subsequent oxidation steps via ent-kaurenoic acid and GA12, catalyzed by the cytochrome monooxygenases *P450-4* and *P450-1*, lead to the formation of GA14 [29,30]. At this stage, specific cytochrome P450 monooxygenases sequentially catalyze the regioselective hydroxylation and oxidation of the ent-kaurene backbone [31]. These defined oxidative modifications are essential for generating the structural diversity and biological activity of final gibberellin products, constituting a critical regulatory node in the pathway [32,33]. GA14 is catalytically converted to GA4 by 20-oxidase *P450-2*. GA7 is converted to GA3 via C13 hydroxylation, a reaction that defines the final step of the pathway. GA4 is then converted by the desaturase (*Des*) to GA7, which undergoes subsequent hydroxylation by the hydroxylase *P450-3* to GA3. A minor portion of GA4 is converted to GA1 by the same hydroxylase *P450-3* [34,35]. The *P450-4* gene encodes a multifunctional cytochrome P450 monooxygenase that catalyzes the three-step oxidation of ent-kaurene to ent-kaurenoic acid [36]. This enzyme mainly catalyzes the 4-step reaction of ent-kaurenoic acid to GA14:7β-hydroxylation, oxidation of the C6 position contracted ring B, 3β-hydroxylation, and oxidation of the C7 position [37]. Among them, 3β-hydroxylation is the most important pathway for metabolic regulation to obtain high yields of GA4/GA7 [38,39]. The complete transformation from GA14 to the final products GA1, GA3, GA4, and GA7 is summarized in Figure 1.

## 3. Metabolic Engineering Strategies to Enhance GA Production

**Figure 2:**
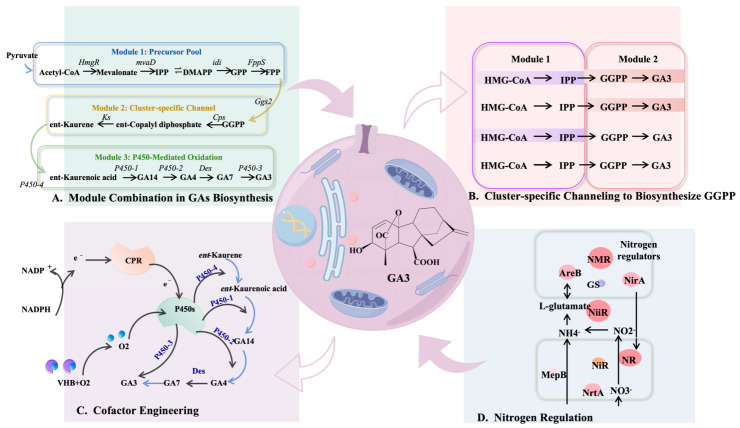
Overview of metabolic engineering strategies for increasing GA production in *F. fujikuroi*. (**A**) The schematic of module combination in GA biosynthesis. (**B**) Cluster-specific channeling to biosynthesize GGPP. (**C**) Cofactor engineering. (**D**) Nitrogen regulation.

Given the crucial role of metabolic homeostasis in GA synthesis, here, we summarize metabolic strategies that can enhance GA production in *F. fujikuroi* (Table 1). Precursor libraries, cluster-specific channeling, and P450-mediated oxidation in the biosynthetic pathway were defined and optimized, respectively (Figure 2A). In addition, nitrogen regulation and histone modification had a strong effect on GA production by *F. fujikuroi* [40]. Nitrogen regulators directly recognize gene cluster sequences for regulation. This regulation requires nitrogen-limiting conditions to activate GA biosynthesis [41,42]. Chromatin structure, which is largely governed by histone modifications, plays a key role in regulating gene expression. The transcriptional activity of most secondary metabolite (SM) gene clusters is closely associated with chromatin conformation, which ranges from open (loosely packed) to closed (densely packed) states depending on histone methylation and acetylation [43].

### 3.1. Pathway Editing and Optimization

Metabolic pathway engineering in *F. fujikuroi* aims to overcome biosynthetic bottlenecks to enhance bioactive GA production, with particular emphasis on optimizing precursor flux [44]. As the foundational route for all terpenoid biosynthesis, the upstream MVA pathway has been systematically engineered to establish an efficient microbial platform [45,46]. A major flux-controlling enzyme in this pathway is *HmgR*, which catalyzes the irreversible, NADPH-dependent conversion of HMG-CoA to mevalonate [47]. To relieve its regulatory limitations, a truncated variant (*tHmgR*) lacking the N-terminal transmembrane domain was expressed, relocating the catalytic domain to the cytosol. This modification improves conformational flexibility and stability, resulting in increased enzymatic activity and a pronounced redirection of metabolic flux. Overexpression of *tHmgR* in *F. fujikuroi* raised the combined titer of GA4 and GA7 to 357.70 mg/L and has previously been shown to increase GA content approximately 2.5-fold relative to the wild-type level. However, systematic studies that specifically quantify the effect of *tHmgR* overexpression solely on GA3 titers remain scarce [29,48].

Subsequent engineering efforts target the supply of universal terpenoid precursors. The enzymes farnesyl diphosphate synthase (*FppS*) and *Ggs2* serve as key nodes directing flux toward diterpenoid backbone synthesis. In particular, overexpression of *Ggs2* has been shown to effectively enhance precursor availability for GA biosynthesis.

The final engineering tier focuses on the GA-specific pathway, notably the diterpene synthase *Cps/Ks*, which catalyzes the committed step converting GGPP to ent-copalyl diphosphate [49]. Its co-expression with *Ggs2* led to a synergistic redirection of metabolic flux, increasing GA3 production relative to the wild type. In the Albermann et al. study, this co-expression strategy elevated the average GA3 yield by approximately 54% relative to the wild type, reaching 315 mg/L under the specific fermentation conditions employed [29,50]. However, kinetic studies reveal that *Cps* activity is susceptible to synergistic substrate inhibition by both Mg^2+^ and its product GGPP, indicating that feed-forward inhibition at this step remains a persistent limitation to flux [31]. This multi-tiered engineering strategy—spanning the upstream MVA pathway, general precursor supply, and the GA-specific step—illustrates how targeted manipulation of rate-limiting enzymes can directly and substantially elevate GA production titers, a principle that has been successfully demonstrated in various terpenoid-producing systems.

### 3.2. Cluster-Specific Channeling to Biosynthesize GGPP

Farnesyl diphosphate (FPP) occupies a central metabolic node, functioning as a key substrate in both the cytoplasmic and mitochondrial branches of isoprenoid biosynthesis. Beyond its role in secondary metabolism, FPP serves as an essential precursor for primary cellular metabolites, including sterols, ubiquinone, heme, dolichol, and prenylated proteins [51].

In fungi, ergosterol is the predominant sterol and a critical structural component of eukaryotic membranes, where it modulates membrane fluidity, permeability, and the activity of membrane-associated proteins [52]. Its biosynthesis proceeds through three major stages: (1) synthesis of mevalonate from acetyl-CoA, (2) conversion of mevalonate to FPP, and (3) the multi-step transformation of FPP into ergosterol. Active ergosterol production supports membrane biogenesis and cellular biomass accumulation. Consequently, the ergosterol pathway constitutes the most direct and major competing route for GA biosynthesis, as both pathways draw from the same intracellular FPP pool. Redirecting metabolic flux toward GA synthesis diverts carbon away from mycelial growth, creating a metabolic burden that can compromise cellular fitness and reduce product yields. Thus, the competitive partitioning of FPP between primary sterol metabolism and specialized GA biosynthesis represents a central challenge in metabolic engineering, necessitating precise optimization of carbon flux to balance growth and production [53].

To redirect metabolic flux from ergosterol biosynthesis toward GA3 production, key strategies focus on partially downregulating early, rate-limiting steps in the sterol pathway. This is commonly achieved through the knockout or repression of genes encoding enzymes such as squalene epoxidase (*ERG1*) or lanosterol synthase (*ERG7*). Disrupting *ERG1* reduces the diversion of FPP into the sterol branch, while inhibiting *ERG7* blocks the cyclization required for the tetracyclic sterol scaffold. These interventions establish a controlled metabolic constraint, redirecting the shared FPP pool toward the engineered GA3 pathway. Crucially, downregulation must be partial to maintain adequate ergosterol for membrane integrity and cellular viability, thereby balancing biomass formation with product titer [54].

To further enhance the efficiency of redirected flux, the spatial-engineering strategy of cluster-specific metabolic channeling has been proposed. Creating a confined metabolic microenvironment can be achieved via protein fusion, synthetic scaffolds, or native interactions that physically couple sequential enzymes. These individual strategies have been shown to facilitate direct substrate transfer from FPP to GA precursors, minimizing the diffusion of intermediates into the competing sterol pathway and insulating GA biosynthesis from metabolic crosstalk. However, the relative contributions of combined strategies versus individual interventions remain largely unexplored, and systematic comparisons are needed to determine whether synergistic effects further enhance GA3 production. Such channeling complements traditional overexpression; while *Ggs2* overexpression alone increased GA production by approximately 150% relative to the wild-type level, co-expression with *Cps/Ks* raised the GA3 titer to 2.55 g/L [29]. This synergy suggests that integrating flux redirection with channeling strategies may partially alleviate the trade-off between product synthesis and growth constraints, indicating an improved balance that supports both cellular viability and product titers (Figure 2B).

### 3.3. Cofactor Engineering

The cytochrome P450 (CYP) system, composed of monooxygenases and their dedicated NAD(P)H-dependent reductases, catalyzes essential monooxygenation reactions. In eukaryotes, the predominant microsomal-type CYP system functions as a complex of membrane-bound P450 and NADPH-dependent cytochrome P450 oxidoreductase (*CPR*) [55]. Within the GA3 biosynthetic pathway, this system conducts sequential oxidative steps, creating a substantial demand for both molecular oxygen and NADPH as a reductant. The cytochrome P450 enzyme system, which functions through the concerted action of P450 monooxygenases and their cognate NADPH-dependent reductases, catalyzes monooxygenation reactions [56].

Consequently, optimizing dissolved oxygen levels and the NADPH supply is critical for efficient GA3 production. To address the high oxygen requirement, heterologous expression of *Vitreoscilla hemoglobin* (*VHB*) is commonly employed. *VHB* scavenges and delivers residual oxygen to terminal respiratory oxidases and oxidative enzymes, thereby sustaining respiratory activity and TCA cycle function, particularly under hypoxic conditions. Simultaneously, the CYP monooxygenases catalyzing the later steps of GA biosynthesis require a significant flux of reducing equivalents. The membrane-associated *CPR* serves as the pivotal electron donor, transferring electrons from NADPH via its FAD and FMN cofactors to the heme iron of P450 enzymes. A synergistic strategy involving co-expression of a codon-optimized *VHB* gene and *CPR* overexpression has therefore been implemented to enhance oxygen delivery and electron flux concurrently [57,58]. Wang et al. refined this combined approach by using the *gpdA* and *tef* promoters to drive dual overexpression of *CPR* and *VHB*. Fermentation studies demonstrated a 31.2% increase in GA3 titer and a 21.9% rise in mycelial biomass. Furthermore, overexpression of *P450-1* increased the total NADP pool by 14% and the NADP^+^/NADPH ratio by 5%, indicating enhanced oxidative capacity and electron transfer efficiency [59,60]. Collectively, these findings indicate that the efficiency of P450-mediated oxidation is governed predominantly by the ratio of NADPH (electron donor) availability to intracellular oxygen concentration [61] (Figure 2C).

### 3.4. Nitrogen Regulation

Nitrogen availability is a primary determinant of GA3 biosynthesis in *F. fujikuroi*, acting through the nitrogen metabolite repression (*NMR*) system. Under nitrogen-sufficient conditions, *NMR* suppresses GA3 production; upon nitrogen limitation, de-repression activates the biosynthetic gene cluster. This section first outlines the core regulatory modules and then systematically describes key factors and their documented effects on GA3 titers [62]. Core signaling and transcriptional activation: Nitrogen deficiency inhibits the Target of Rapamycin (TOR) kinase pathway, relieving repression of the global regulator *AreA* and activating *AreB*. The resulting *AreA*/*AreB* heterodimer translocates to the nucleus and binds GATA/TATC motifs in target gene promoters, directly stimulating GA3 biosynthesis [63].

Nitrate and ammonium assimilation pathways: Nitrate utilization is transcriptionally regulated by NirA (nitrate-specific factor) and *AreA*. In the presence of nitrate, NirA activates genes for nitrate assimilation (*NiaD*, *NiiA*, and *NrtA*). Fungi also possess an *AreA-* and NrtA-independent nitrate sensing mechanism that relies on nitrate reductase (NR) activity, ensuring precise transcriptional responses that indirectly influence metabolic fluxes toward secondary metabolism [64]. Ammonium assimilation proceeds via NADP^+^-dependent glutamate dehydrogenase (GdhA) and glutamine synthetase (GS); GS activity is feedback-inhibited by NH_4_^+^ and glutamate. Cellular glutamine levels are tightly regulated through *AreA*-mediated *NMR*, fundamentally restricting GA3 biosynthesis under nitrogen-sufficient conditions [65].

Fine-tuning via MeaB and NmrA: The bZIP protein MeaB mediates *NMR* by activating transcription of the co-repressor NmrA, which inhibits *AreA*. Under nitrogen starvation, inhibition of MeaB modestly upregulates GA3 biosynthetic genes and specific nitrogen transporter genes. Notably, nitrogen repression signaling requires coordinated input from both TOR kinase and MeaB [66,67] (Figure 2D).

Key engineering targets and yield outcomes: *AreA*, the central GATA-type activator for alternative nitrogen sources, supports mycelial growth and directly induces expression of the GA3 biosynthetic gene cluster following nitrogen assimilation [68]. *Lae1* is essential for GA3 production; its overexpression relieves nitrogen repression and activates the GA3 gene cluster. Overexpression of *AreA* and *Lae1* individually yielded GA3 titers of 2.51 g/L and 2.35 g/L, respectively [69]. Furthermore, nitrogen regulatory factors can indirectly influence histone modification [70]. A sophisticated metabolic engineering approach incorporated this knowledge by co-overexpressing *AreB*, histone acetyltransferase *Hat1*, and adaptor protein *Ada3* under dynamic promoter regulation. This strategy achieved a final GA3 titer of 3.16 g/L, enhanced carbon/nitrogen metabolic flux, increased biomass, balanced cofactor and oxygen supply via a nitrogen-responsive promoter, and prevented terpenoid byproduct accumulation [71].

**Table 1:** Metabolic engineering strategies of GA3 production in *F. fujikuroi*.

Function Module	Gene	Gene ID	Engineering Strategy ^a^	GA3 Titer (g/L)	Function Annotation	References ^b^
Precursor supply	*HmgR*	FFUJ_04000	OE: *HmgR*	2.23	Key rate-limiting enzyme in the MVA pathway, catalyzing the generation of terpene skeleton precursors	[58]
	*Cps/Ks*	FFUJ_14336	OE: *Cps/Ks*	2.18	Catalyzed two-step cyclization of GGPP to produce endogenous shellacene	[58]
	*FppS*	FFUJ_03086	OE: *FppS*	2.14	Catalyzes the formation of FPP from GPP and IPP	[58]
Cofactor engineering	*VHB*	FFUJ_01089	OE: *VHB*	2.20	Can bind to oxygen and increase oxygen delivery in the periplasmic space of the cell	[58]
	*CPR*	FFUJ_04716	OE: *CPR*	2.25	Providing electrons to the cytochrome P450 monooxygenase system	[58]
Nitrogen regulation	*AreB*	FFUJ_05048	OE: *AreB*	2.45	Nitrogen catabolic enzyme regulatory protein	[68]
	*AreA*	FFUJ_06143	OE: *AreA*	2.51	Similar to *AreB*, forming part of the nitrogen regulation system	[64]
	*Hat1*	FFUJ_03208	OE: *Hat1*	2.40	Catalytic component of the histone acetylase B (HAT-B) complex	[68]
	*Ada3*	FFUJ_00496	OE: *Ada3*	2.12	Forms complexes with Gcn5 and Ada2 and participates in histone acetylation modification-related processes	[68]
	*AreB*/*Hat1*/*Ada3*	FFUJ_05048, FFUJ_03208, FFUJ_00496	o-OE: *AreB*/*Hat1*/*Ada3*	3.16	Multi-target nitrogen regulation combining transcriptional regulator, histone acetyltransferase, and adaptor protein	[71]
Global regulators	*Lae1*	FFUJ_00592	OE: *Lae1*	2.35	Methyltransferase that performs automethylation and controls the expression of the GAs gene clusters	[64]

^a^ Abbreviations: OE, overexpression; ^b^ data for *Ggs2*, *HmgR*, *Cps/Ks*, *FppS*, *VHB*, and *CPR* are from a single study, in which each gene was individually overexpressed under the same experimental conditions.

### 3.5. Histone Modification

**Figure 3:**
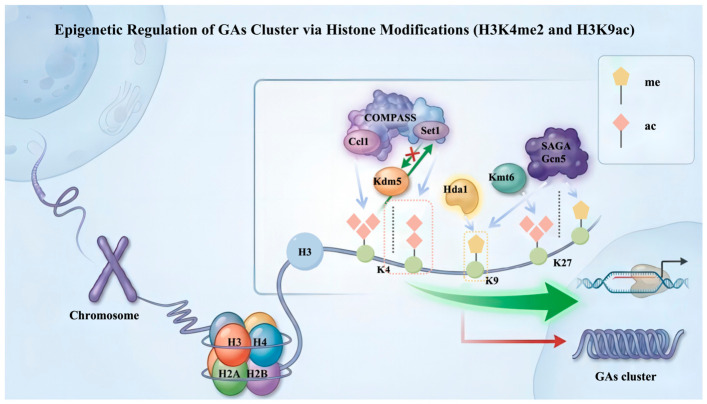
Histone modifications associated with GA3 biosynthesis in *F. fujikuroi*. Reported histone modifications involved in GA3 biosynthesis include H3K4me2 and H3K9ac, which are highly correlated with transcript levels of GA cluster genes. HDACs: histone deacetylases; SAGA: Spt-Ada-Gcn5 acetyltransferase.

Histone modifications play a key role in regulating secondary metabolism in filamentous fungi. The entire GA biosynthesis process is regulated through a combination of extranuclear protein interactions and signal transduction pathways, as well as intranuclear transcription factor activity [72]. Most histone modifications are reversible and dynamic. In fungi, secondary metabolite biosynthetic gene clusters are often controlled by chromatin remodeling, with histone acetyltransferases (HATs), deacetylases (HDACs), methyltransferases, and proteins involved in heterochromatin formation playing critical regulatory roles [73]. Gcn5, a member of the Gcn5-related N-acetyltransferase (GNAT) family and a key component of the Spt-Ada-Gcn5 acetyltransferase (SAGA) complex, is responsible for acetylating lysine residues on histone H3. In *F. fujikuroi*, the Gcn5/Ada2/Ada3 complex is essential for the acetylation of H3 at lysine residues K4, K9, K18, and K27 [74]. Among these, acetylation at H3K9 is specifically associated with activation of the GA biosynthetic gene cluster. Consistent with this, HPLC analysis showed that deletion of Gcn5 significantly downregulated GA cluster gene expression and reduced metabolite production [75]. HDACs are widely conserved post-translational modifiers in filamentous fungi. The acetylation dynamics are governed by two enzyme groups: (1) HATs, which transfer acetyl groups from acetyl-CoA to lysine ε-amino groups, and (2) HDACs, which remove these modifications, thereby maintaining a precise balance between acetylated and non-acetylated lysine states [76]. The deletion of two Zn^2+^-dependent HDAC-encoding genes (*Ffhda1* and *Ffhda2*) indicated that *FfHda1* and *FfHda2* can regulate secondary metabolism. This resulted in a 64% and 25% reduction in HDAC activity, respectively. H3K4 methylation was found in the transcribed euchromatic region of *F. fujikuroi* [77]. It was also found to be significantly associated with actively expressed genes in *Aspergillus nidulans* and *Fusarium graminearum.* These findings suggest that most secondary metabolite gene clusters in *F. fujikuroi* are indirectly regulated and are not direct targets of *Set1* or *Kdm5* [78,79]. H3K4 can be mono-, di-, or trimethylated, a modification implicated in the activation or silencing of gene clusters. Specifically, H3K4 dimethylation (H3K4me2) levels positively correlate with GA3 biosynthesis, and transcriptional analyses have shown that elevated H3K4me2 is associated with increased transcript levels of *P450-4* and *P450-2*. Set1/COMPASS binds to the C-terminal domain (CTD) of RNA polymerase II to modulate H3K4 methylation levels and distribution [80]. *Ccl1,* a subunit of the COMPASS complex, catalyzes H3K4 methylation. Although H3K9 acetylation upregulation increased expression of the GA gene cluster, GA3 production was halved in *ccl1*-deficient strains, indicating that *ccl1* regulates H3K4 genome-wide trimethylation (H3K4me3) [81]. Furthermore, *Set1* functions as an activator and *Kdm5* as a repressor of H3K4me3. These two regulators antagonistically control both global H3K4me3 levels and the expression of *ABA1*, a major nodule-specific transcription factor gene in *F. fujikuroi*. Fermentation analyses revealed that deletion of *Kdm5* caused transcriptional instability in several GA biosynthetic gene clusters and decreased total GA production, whereas *Kdm5* overexpression enhanced GA titers. It has been established that *Kdm5* demethylates H3K4me3 in vivo. The residual H3K4me2/me1 marks may be further removed by an Lsd1-type amine oxidase in *F. fujikuroi* [74] (Figure 3). Of note, the above studies focused on elucidating epigenetic mechanisms rather than optimizing fermentation performance and thus provided only qualitative or semi-quantitative assessments of GA3 production. Nonetheless, these findings establish a critical foundation for future engineering of chromatin modifiers to enhance GA biosynthesis.

In summary, the five strategies above exhibit distinct trade-offs in scalability, metabolic burden, and overall feasibility (Table 2). Single-target modifications offer better scalability and lower burden, whereas multi-target strategies yield higher titers at the expense of increased complexity. Pathway editing and cluster-specific channeling effectively enhance precursor supply but impose constitutive burden that limits scalability. Cofactor engineering provides a low-burden, scalable approach, yet the titer gains are marginal. Histone modification enables cluster-wide epigenetic regulation, but its yield effect remains unquantified and industrial feasibility remains low. Beyond these individual strategies, a recent multimodular engineering approach combining fatty acid biosynthesis reinforcement, acetyl-CoA flux augmentation, redox cofactor optimization, and *Lae1* overexpression achieved 2.58 g/L GA3 in strain OE: *Lae1-AGP3*, which was further elevated to 2.86 g/L by exogenous fatty acid supplementation, underscoring the synergistic potential of integrating metabolic module engineering with bioprocess optimization [82]. Future priorities include marker-free editing, dynamic regulation, and epigenetic stability assessment.

Single-module engineering (precursor/cofactor) achieves marginal increments, nitrogen regulation yields the highest titers but is contingent upon nitrogen-limiting conditions, and epigenetic modulation remains quantitatively uncharacterized; only their combinatorial integration enables maximum yield improvement while mitigating metabolic burden and industrial feasibility constraints (Table 3).

## 4. New Tools and Applications in Metabolic Engineering

### 4.1. CRISPR Gene Editing in F. fujikuroi

The CRISPR/Cas9 system mediates DNA editing through the Cas9 endonuclease, which is directed by a dual-RNA complex (crRNA and tracrRNA) to generate DNA double-strand breaks (DSBs). For application in eukaryotes, Cas9 is typically fused to a nuclear localization signal (NLS) and guided by a chimeric single-guide RNA (sgRNA) [83,84].

**Figure 4:**
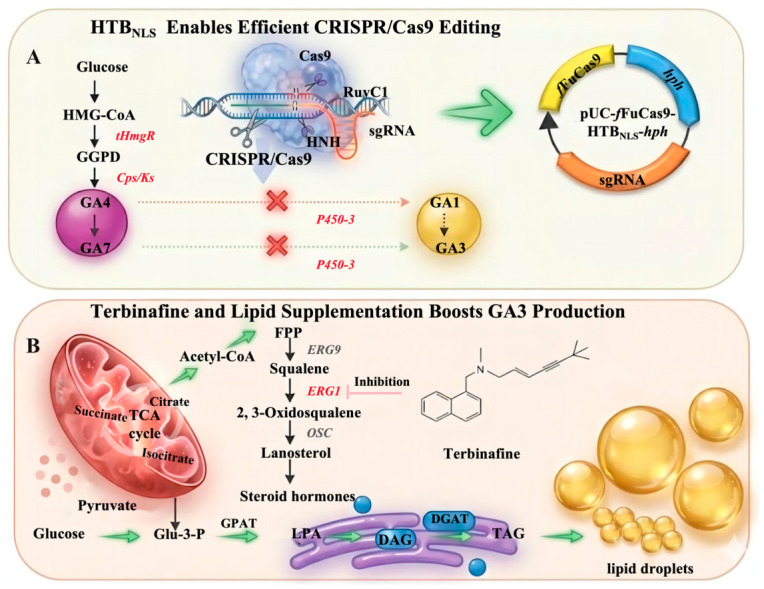
New tools in metabolic engineering. (**A**) The HTB_NLS_ nuclear localization signal enables efficient CRISPR/Cas9-mediated gene editing in *F. fujikuroi*. (**B**) Supplementation with terbinafine and lipid compounds effectively boosts GA3 production.

However, establishing efficient CRISPR/Cas9 editing in the non-model fungus *F. fujikuroi* encounters several specific constraints: (1) inefficient nuclear import of Cas9, attributable to the poor functionality of canonical nuclear localization signals (SV40_NLS_), indicating divergent importin-mediated transport; (2) suboptimal sgRNA transcription, as heterologous RNA polymerase III (Pol III) promoters frequently exhibit low activity, necessitating the identification of native regulatory elements; and (3) a dominant non-homologous end joining (NHEJ)-mediated DNA repair landscape that complicates precise homology-directed repair (HDR), a common characteristic of filamentous fungi [85,86] (Figure 4A).

To circumvent these barriers, an optimized editing platform was developed through targeted engineering: screening identified fungal-optimized nuclear localization signals (NLSs), with histone H2B-derived HTB_NLS_ and Velvet-derived VEL_NLS_ achieving mutation efficiencies of 4% and 41.7% [87,88]; concurrently, endogenous, high-activity RNA Pol III promoters were utilized, with the native U6 snRNA and particularly the multi-copy 5S rRNA locus (*Ff 5SrRNA*) yielding the highest editing rates by ensuring promoter compatibility and providing a gene dosage effect [89]. The integration of an effective NLS (HTB_NLS_) with sgRNA expression driven by the robust native 5S rRNA promoter established a highly efficient genome-editing platform in *F. fujikuroi*, achieving editing efficiencies up to 79.2% [86,90].

The optimized system enables rational manipulation of the GA biosynthetic gene cluster (GBC) by facilitating (1) multiplexed knockout of competing metabolic branch genes to funnel flux toward GA precursors; (2) precise promoter engineering of core pathway genes; and (3) iterative combinatorial editing for combinatorial strain engineering. This shifts the paradigm from random mutagenesis to systematic pathway refactoring, directly addressing yield and specificity limitations [91].

### 4.2. Promoter Engineering

A promoter is a regulatory DNA sequence that binds transcription factors and initiates transcription. Promoter engineering designs synthetic cis-regulatory elements with tailored functionalities absent in native promoters. These engineered constructs typically exhibit enhanced strength, shorter length, reduced sequence repetition, and support layered gene regulation, thereby driving higher enzyme expression than natural counterparts to increase flux through metabolic pathways [92]. Strategic deployment of synthetic promoters effectively optimizes pathway performance and improves bioproduct yield [93]. To address the limited availability of well-characterized genetic regulatory elements in *F. fujikuroi*, Qi et al. constructed a library of 20 candidate promoters based on transcriptomic data, assessing their strength using a β-glucosidase reporter system. The promoter P10594 demonstrated the highest and most stable activity. Overexpression of isopentenyl diphosphate isomerase (*IDI*) under its control successfully increased *IDI* transcript levels and redirected metabolic flux, raising GA3 titer by 17.1% (from 0.76 to 0.89 g/L) in shake-flask culture [94,95]. This validates the use of strong endogenous promoters to overcome pathway bottlenecks. Current engineering primarily employs two promoter types: strong constitutive promoters (*PgpdA* and *PtrpC*) for balancing multi-enzyme expression and inducible systems (*PniiA*) to separate growth and production phases [96]. While constitutive expression ensures constant enzyme supply, it may burden growth; inducible promoters offer dynamic control but require precise regulation. Future efforts must advance from screening natural variants to the rational design of synthetic promoter libraries. This will enable precise, dynamic, and industrially robust control over metabolic pathways, moving beyond the current reliance on a limited set of native regulatory elements. The lower titer (0.89 g/L) in shake flasks reflects the laboratory strain and cultivation scale; the difference from other strategies primarily stems from strain background and fermentation conditions rather than promoter performance.

### 4.3. Targeting Technology for Small-Molecule Compounds

Exogenous small molecules serve as pivotal chemical modulators in microbial metabolic engineering, functioning as enzyme- or pathway-specific activators/inhibitors to redirect metabolic flux and enhance fermentation performance, as demonstrated in *F. fujikuroi* for GA3 production [97,98]. Unlike empirical chemical supplementation, the systematic “small-molecule compound-based targeting technology” recently established for *F. fujikuroi* integrates transcriptomics analysis, in vivo genetic engineering, and scale-up validation as a rational tool for strain improvement.

A primary strategy involves the chemical inhibition of competing biosynthetic pathways to enrich precursor availability for GA3 synthesis. For example, terbinafine, an inhibitor of squalene monooxygenase, a key enzyme in sterol biosynthesis, blocks this membrane-associated enzyme, depletes ergosterol, causes intracellular squalene accumulation, and redirects metabolic flux toward GA3 biosynthesis, elevating its titer to 1.08 g/L [99,100]. Genetic evidence supports this approach: downregulating squalene biosynthesis via promoter truncation similarly enhanced GA3 yield by reducing competitive carbon diversion. In contrast, inhibitors of the upstream MVA pathway, such as simvastatin or lovastatin, limit the supply of central isoprenoid precursors (IPP/DMAPP) and consequently impair GA3 production [101] (Figure 4B).

Beyond pathway-specific inhibition, chemical modulators can also alleviate cellular stress or modulate global regulation. α-Tocopherol, for instance, reduces reactive oxygen species (ROS) burden via direct scavenging and indirect upregulation of antioxidant enzymes, thereby improving cellular fitness and raising squalene accumulation by 63.2% [99]. Supplementation with lipid substrates such as waste cooking oil at the initiation of fermentation can likewise stimulate fatty acid synthesis and enhance GA3 biosynthesis. Furthermore, epigenetic modulators like the histone deacetylase inhibitor Trichostatin A (TSA) not only increase GA production but can also activate otherwise silent secondary metabolite gene clusters.

In summary, the rational deployment of small-molecule modulators—through direct pathway inhibition, mitigating physiological constraints, or epigenetic remodeling—enables precise and adjustable control of metabolic flux. This chemical approach, complementary to genetic engineering, provides a refined strategy to optimize GA3 yield and orchestrate the secondary metabolome of *F. fujikuroi*.

### 4.4. Genome-Scale Metabolic Model

Genome-scale metabolic models (GEMs) are systems biology frameworks that computationally reconstruct an organism’s complete metabolic network based on genome annotations and biochemical data. Structured as a stoichiometric matrix comprising mass-balanced reactions and metabolites, GEMs allow for the prediction of metabolic fluxes and growth phenotypes using constraint-based optimization approaches [102]. GEMs function as in silico representations of metabolic networks, integrating genes, reactions, and their catalytic relationships within a stoichiometric matrix that encodes gene–protein–reaction (GPR) associations. This framework enables constraint-based simulation for predicting metabolic fluxes and conducting systems-level metabolic analyses [103]. The utility of GEMs for phenotype prediction and metabolic engineering has been demonstrated across diverse organisms. For instance, GEM-guided optimization of 20 target proteins in *Escherichia coli* (*E. coli*) enabled the rational engineering of lysine biosynthesis, achieving a titer of 95.7 ± 0.7 g/L [104]. Similarly, the model iTN656 elucidated nutrient essentiality in *Limosilactobacillus reuteri* (*L. reuteri*), identifying critical mono- and di-nutrient pairs necessary for growth [105].

In *F. fujikuroi*, the construction of the GEM iCY1235 has provided a pivotal platform for elucidating and engineering the GA biosynthetic pathway. The model identified mannitol as a favorable carbon source that enhances acetyl-CoA flux and oxygen uptake, thereby promoting GA3 synthesis. Subsequent application of the OptForce algorithm pinpointed 20 reaction targets directly associated with GA metabolism. Experimental validation confirmed that overexpression of two key genes (FFUJ_02053 and FFUJ_14337) increased GA3 titers by 37.5% and 75%, respectively, translating model predictions into actionable metabolic engineering strategies [106]. Despite these advances, the development and application of fungal GEMs are hindered by limited genetic tools, including a scarcity of well-characterized promoters and efficient transformation systems. To improve predictive accuracy for the GA pathway, we constructed a high-quality GEM for *F. fujikuroi* by integrating multi-omics data (transcriptomic, proteomic, and metabolomic). Dynamic flux analysis of this model revealed two major systemic bottlenecks in GA biosynthesis: low branching efficiency at FPP and cofactor (NADPH/ATP) supply limitation. These results highlight the utility of GEMs for diagnosing flux constraints and guiding strategies to optimize GA production.

## 5. Conclusions and Perspectives

GAs, especially those microbially produced by *F. fujikuroi*, have vast potential in terms of their application in agriculture and food [107]. GAs exhibit broad bioactivity in regulating plant growth and developmental processes [108,109].

Large-scale industrial production of GA3 remains restricted by low productivity. This review explores and discusses metabolic engineering strategies to overcome this limitation. With the continuous advancement of molecular biology, the synthetic pathways of GAs and the functions of key enzyme genes have been extensively studied. Researchers have employed multiple strategies to enhance GA production, primarily including pathway editing, constructing cluster-specific pathways for GGPP biosynthesis, optimizing cofactors, regulating nitrogen metabolism factors, and utilizing histone modifications [110,111].

However, current production levels still fall short of meeting industrial demand, leaving room for improvement. Extensive and in-depth research should be conducted, encompassing systems metabolic engineering, regulatory element engineering, and transporter engineering. Metabolic engineering strategies and genetic tools facilitate the development of high-yielding GA-producing strains. Genome editing tools, promoter engineering, small-molecule compound targeting, GEMs, and functional genomics enhance our understanding of key rate-limiting steps and systemic bottlenecks. In *F. fujikuroi*, mining efficient enzymes, increasing the activity of key enzymes by metabolic engineering, overexpression of key enzymes, deletion of the negative regulatory genes, cofactor supply, promoter optimization, and fermentation conditions are common strategies to improve the yield of GAs. The integration of systems biology and synthetic biology tools provides powerful support for overcoming biosynthetic bottlenecks. In *F. fujikuroi*, the CRISPR-Cas9 system enables gene editing via Cas9 protein expression without affecting growth. Transcriptome sequencing analyzes metabolic flux under strong promoters to identify key rate-limiting steps. Metabolic flux is regulated using small-molecule targeting technology and the iCY1235 model. Biosynthetic research has paved the way for enhancing GA production. Optimizing auxiliary factor supply and dynamically regulating precursor allocation represent promising strategies for enhancing GA production while maintaining cellular adaptability [112]. Unlike previous reviews, this work integrates pathway engineering, regulatory mechanisms, and genetic tools in *F. fujikuroi*, evaluating beneficial and detrimental strategies (*FppS* overexpression), identifying mechanistic bottlenecks (feedback inhibition and metabolic load), and proposing combined applications of optimized CRISPR/Cas9, GEMs, and chemical modulators to provide practical guidance for strain engineering.

However, industrial translation faces major bioprocess hurdles. Oxygen transfer limitation reduces GA3 titers upon scale-up [71]. Morphological instability and shear sensitivity impair mixing and oxygen supply, while strain degeneration driven by oxidative stress causes productivity loss [113]. These constraints increase purification costs and lower volumetric productivity. Without mitigation-shear-tolerant strains, morphology control, oxygen optimization, and resin-based purification, even advanced metabolic engineering may fail economically.

Advancing GA biosynthesis in *F. fujikuroi* necessitates an integrated strategy spanning multiple technological frontiers. Tool development is foundational, requiring expansion of the genetic toolkit with modular regulatory components and efficient delivery systems to enable precise CRISPR/Cas9 genome editing, promoter engineering, and epigenetic modulation for fine-tuned transcriptional control of the GA biosynthetic gene cluster. Systems-level analysis, achieved through the integration of multi-omics data into context-specific GEMs, is essential for identifying systemic bottlenecks such as metabolic flux partitioning at the FPP node and limitations in cofactor (NADPH/ATP) supply, thereby informing rational strain design [114]. Pathway and cofactor engineering must extend beyond core GA biosynthetic genes to encompass dynamic precursor pathway assembly, rerouting of competing metabolic fluxes, and implementation of efficient cofactor regeneration systems. Computational and automated design, leveraging artificial intelligence and machine learning for in silico enzyme and metabolic pathway optimization—combined with high-throughput screening and automated strain construction—will expedite the design–build–test–learn cycle for generating high-performance production strains.

Nevertheless, industrial translation of these engineered strains must overcome challenges in scale-up, downstream processing, and regulation. Fermentation scale-up requires process analytical technology and quality-by-design principles, while downstream purification remains a major cost driver. Encouragingly, the European Food Safety Authority (EFSA) recently concluded that neither GA4/GA7 are endocrine disruptors, offering a favorable regulatory path. Future efforts should focus on low-cost substrates, continuous fermentation, integrated purification, and early regulatory engagement to accelerate commercialization [115]. This removes a major regulatory hurdle for EU re-approval and supports potential classification as low-risk substances.

Industrial GA production must overcome oxygen transfer limitation, morphological instability, shear sensitivity, high purification costs, and strain degeneration. Solutions include microparticle-assisted morphology control, shear-tolerant strains derived via adaptive laboratory evolution (ALE), cost-effective adsorbents, and routine stability monitoring. Emerging tools—AI-assisted metabolic modeling, ALE, dynamic biosensors, and synthetic organelle engineering—offer complementary advances. Weng et al. raised GA3 titers to 2.86 g/L via modular engineering and fermentation optimization [82]. Sun et al. reviewed protein self-assembly systems for enhanced metabolic channeling [116]. Integrating these strategies will accelerate the development of robust *F. fujikuroi* cell factories for industrial GA4/GA7 production.

## Figures and Tables

**Table 2:** Comparison of metabolic engineering strategies for GA3 biosynthesis in *F. fujikuroi*.

Strategy ^a^	Key Features	Advantages	Limitations
Pathway editing (co-OE of *Ggs2* and *Cps/Ks*)	Directly enhances precursor supply	Effective titer increase	Constitutive overexpression imposes metabolic burden and limits scalability
Cluster-specific channeling	Spatial engineering	May alleviate growth–production trade-off	Requires co-expression strategies
Cofactor engineering (co-OE of *CPR* and *VHB*)	Improves oxygen and NADPH supply	Low metabolic burden; high scalability	Gain is marginal
Nitrogen regulation (single target: *AreA* OE)	Cluster-specific control	High feasibility; low burden; high stability	Requires nitrogen-limited media
Nitrogen regulation (multi-target: co-OE of *AreB*, *Hat1*, and *Ada3*)	Combines regulatory, epigenetic, and adaptor proteins	Inducer-free	Increased complexity and metabolic burden
Histone modification (H3K4me2/H3K9ac)	Enables cluster-wide epigenetic upregulation	Potential for simultaneous gene cluster activation	Low industrial feasibility

^a^ Abbreviations: OE, overexpression.

**Table 3:** Functional modules of metabolic engineering for GA3 production in *F. fujikuroi*.

Functional Module	Representative Targets	Engineering Strategy	Function Description
Precursor supply	*Ggs2*, *Cps/Ks*, *HmgR*, *FppS*	Overexpression	Enhances flux toward GGPP and diterpenoid backbone
Cofactor supply	*CPR*, *VHB*	Co-overexpression	Improves oxygen delivery and NADPH supply for P450-mediated oxidations
Nitrogen regulation	*AreA*, *AreB*, *Lae1*	Overexpression	Relieves nitrogen repression and activates GA cluster transcription
Epigenetic regulation	*Set1*, *Kdm5*, *HDACs*	Deletion or overexpression	Modulates chromatin accessibility and cluster gene expression

## Data Availability

No new data were created or analyzed in this study. Data sharing is not applicable to this article.
